# The combination of dendritic cells-cytotoxic T lymphocytes/cytokine-induced killer (DC-CTL/CIK) therapy exerts immune and clinical responses in patients with malignant tumors

**DOI:** 10.1186/s40164-015-0027-9

**Published:** 2015-11-10

**Authors:** Ying Wang, Zenghui Xu, Fuping Zhou, Yan Sun, Jingbo Chen, Linfang Li, Huajun Jin, Qijun Qian

**Affiliations:** Laboratory of Gene and Viral Therapy, Eastern Hepatobiliary Surgery Hospital, the Second Military Medical University of Chinese PLA, Shanghai, 200438 China; Department of Biotherapy, Eastern Hepatobiliary Surgery Hospital, the Second Military Medical University of Chinese PLA, Shanghai, 200438 China

**Keywords:** DC-CTL/CIK immunotherapy, Serological tumor markers, Immune indicators, Quality of life, Tregs

## Abstract

**Background:**

The clinical trials using immunotherapy have been performed for the treatment of variety of malignant tumors. However, large-scale meta-analysis of combined DC-CTL/CIK therapy on immune and clinical response in patients has not been well studied yet. The purpose of this study is to investigate the role of DC-CTL/CIK therapy and evaluate the changes of immune indicators and tumor serological markers both at an individual level and at a system level, which is an important basis for immunotherapy as well as prognosis estimation.

**Methods:**

Three cohorts were designed to estimate therapeutic effects on patients with malignant tumors. Tumor serological markers were detected pre- and post-treatment by immunoradiometric methods using commercially available diagnostic kits. Lymphocyte subsets were identified by flow cytometry. The quality of life was assessed by EORTC QLQ-C30 questionnaire.

**Results:**

In this study, we found out that Tregs was significantly reduced after transfusion of DC-CTL/CIK cells companied by decreasing serological tumor markers including AFP, CA199 and CA242 in primary liver cancer and CA724 in gastric cancer. A system-level analysis showed that lower percentages of Tregs were detected in patients with long-lasting courses of immunotherapy. Strikingly, a tumor progression indicator, myeloid-derived suppressor cells (MDSC), was dramatically decreased in patients after DC-CTL/CIK treatment. These results suggested that DC-CTL/CIK therapy improves immune functions and the quality of life post-treatment versus pre-therapy, indicating that DC-CTL/CIK therapy might block the deterioration of invasive cancers in these patients.

**Conclusions:**

This study demonstrated that DC-CTL/CIK therapy could reduce Tregs, MDSCs, and several crucial serological tumor markers in particular tumors, and improve the function of T cells immune systems and the quality of life in patients with malignant tumor.

**Electronic supplementary material:**

The online version of this article (doi:10.1186/s40164-015-0027-9) contains supplementary material, which is available to authorized users.

## Background

Malignant tumors are refractory diseases that cause death and high economic burden in the world. Traditional therapeutic regimens such as surgery, radiation, or chemotherapy are usually accompanied by adverse side-effects. Nowadays, immunotherapy has emerged as an effective treatment for patients with malignant tumors. It has been broadly applied in clinical trials for the treatment of various malignant tumors. Lots of trials have demonstrated that the therapeutic use of DC vaccines, CTL cells and CIK cells in the improvement of cancer therapy and showed promising outcomes of DC-CTL/CIK therapy alone or combined with conventional therapies [1, 2]. In previous reports, several studies have focused on improving curative effect and evaluating the safety and efficacy of immunotherapy [3–8], founding that DC vaccines combined with CIK cells might have a potential to prevent tumor recurrence, increase progression-free survival rates, and improve the quality of life for cancer patients. Moreover, the side effects and toxicity of immunotherapy were mild and easily controllable.

DC-CTL/CIK treatment have displayed encouraging results in tumor therapy, however, several critical issues remain to be solved. First of all, the study of immunotherapy in solid tumors is still rare although several clinical trials had been carried out in the treatment of metastatic renal cell carcinoma, colorectal cancer and other malignancies [7, 9]. The sample sizes for most of trials are small. Secondly, there is still no reliable biomarker for evaluating clinical responses and monitoring prognosis of DC-CTL/CIK therapy. The indicators such as CD3, CD4, and CD8 have been selected as common indicators to assess the efficacy of immunotherapy. The changes of those indexes were statistically significant in patients post-treatment versus pre-therapy, however, whether this or other biomarkers correlate with clinical outcome remain less explored [10–12]. Thirdly, there is still no standard cell preparation system of DC-CTL/CIK therapy. A lot of robust cell culture regimens have been established, however, different laboratories performed variously in cells culture regimens such as numbers of infused cells, types of additional cytokines, concentration and incubation time [13–16], which could have an impact on the efficacy of DC-CTL/CIK therapy. In the previous studies, Qu et al. [15] reported the number of CIK cells cultured in vitro increased to 14.3 ± 2.6 times at 12 days culture period, and this number could reach to 20.5 ± 3.2 times when CIK cells was co-cultured with DC cells. Pan et al. [14] and Tao et al. [16] depicted that the main functional properties of CIK cells might be limited by Tregs. Theoretically, improvement of DC-CTL/CIK culture strategy and depletion of Tregs could bring benefit to tumor therapy. Thus, a more standardized and efficient cell production process is needed to be well-studied.

Therefore, in the present study, we designed three cohorts, totally including 83 patients, to investigate therapeutic effect of DC-CTL/CIK therapy in patients with malignant solid tumors. To identify the essential features overall and investigate the therapeutic role of DC-CTL/CIK therapy, we attempted to assess several common serological tumor markers and immune indicators across a wide range of tumor types at an individual level. And then, at a systematic level we performed to analyze the correlation between changes of Treg cells and prognosis and to assess the long-term quality of life. Additionally, a novel tumor progression indicator, myeloid-derived suppressor cells (MDSC), was also being approached preliminarily in this study.

## Methods

### Patients

We consecutively analyzed cohorts of 83 patients in three subgroups. The first subgroup (cohort 1) contained 60 patients with detailed the clinicopathologic information about sex, age, Tumor Classification, tumor-node-metastasis (TNM) stage, adjuvant therapeutic strategies, serological tumor markers detection, immune indicators detection and follow-up of prognosis, who were from Biotherapy Department of Eastern Hepatobiliary Surgery Hospital, received DC-CTL/CIK therapy from February 2012 to March 2014 (25 patients with primary liver cancer, 10 patients with cholangiocarcinoma, 11 patients with lung cancer, 7 patients with gastric cancer and 7 patients with colon cancer). In Cohort 2, 14 patients with negative for all serological tumor indexes were reviewed. Cohort 3 included nine patients for MDSC detection. The peripheral blood samples were accessed in Department of Biotherapy, Eastern Hepatobiliary Surgery Hospital, the Second Military Medical University (China). All patients signed informed consents.

### DC-CTL/CIK cells culture regimen

Peripheral blood mononuclear cells (PBMCs) were harvested by blood cell separator (FreseniusKabi, Germany). About 1 × 10^9^–3 × 10^9^ PBMC cells/40–60 ml white cells were collected from each patient, and were cultured in AIM-V cell culture solution (Gibco, America) for 2 h. The adherent cells were initiated into culture for DC maturation by 100 ng/ml GM-CSF (PeproTech, America) and 100 ng/ml IL-4 (PeproTech, America) in AIM-V cell culture solution. Part of the non-adherent cells were stored at −80 °C and thawed for co-cultivation, and the rest were applied for inducing CIKs.

On Day 6, the recombinant non-proliferative adenovirus (serotype 35) carrying the expression cassettes of tumor-associated antigens (TAAs) were used to infect the DC cells with a multiplicity of infection (MOI) of 5. After 24 h cultivation, IL-1β (25 ng/ml; Novoprotein, China) and TNF-α (100 ng/ml; Novoprotein, China) were added into the culture for DC-maturation for another 24 h.

On day 8, about 2 × 10^6^ DC cells were harvested and co-cultured with T cells (about 4 × 10^7^ cells) at a DC/T cell ratio of 1:20 for another 4 days to induce antigen-specific CTL cells which were stimulated with CD3 monoclonal antibody (mAb) (50 ng/ml; Novoprotein, China) pre-coated onto plastic plates and amplified by IL-2 (500 IU/ml; Novoprotein, China). The rest cells were applied as DC vaccine. The applied TAAs included p53, survivin, hTERT and additional AFP/CEA for AFP/CEA positive patients.

Additionally, CIKs were cultured in 4 × 40 Ml serum-free medium supplemented with 1000 U/mL IL-2, 5 μg/mL CD3 monoclonal antibodies, 12.5 μg/ml RetroNectin (Novoprotein, China) and 1000 U/mL IFN-γ (Novoprotein, China).

### Cell collection and centrifugation

Cells were harvested on day 8, transferred from medium bags into centrifuge bottles, and centrifuged at 1500 rpm for 5 min, removed the supernatant by aspiration, washed twice by adding normal saline, then removed the supernatant and re-suspended the cell pellet in 300 ml normal saline and 500 μl IL-2. We transferred cells into transfer bag, heated seal the bag and prepared for clinical infusions. The protocol of cell collection and centrifugation on day 10 and day 12 is the same as day 8.

### Preparation for infusions

The infused cells were cultured for detecting levels of bacteria, fungus and endotoxin by using BacT/ALERT^®^ 3D instrument (Biomerieux, America) and and PREVI^®^ Color Gram (Biomerieux, America).

In total, the final volume is 1200 ml (300 ml/per bag × 4 bag = 1200 ml). Promethazine is used to treat allergy symptoms 10 min before starting the treatment. The usual dose is 12.5 mg by intramuscular injection. After the infusion, Sodium bicarbonate is used to make the urine more alkaline to prevent kidney failure. During the procedure of infusion, the drop speed in the first 15 min is 30 drops/min. If patients have no adverse reaction after 15 min, we adjust the drop speed at 80 drops/min.

### Treatment schedules

For each treatment, patients were treated with 3 intravenous infusions. About 1 × 10^7^ DC vaccine, 1 × 10^9^ CIKs and both DC vaccine and CTLs (about 1 × 10^9^ cells) were infused intravenously on day 8, day 10 and day 12 respectively.

### Detection of immune indicators by flow cytometry

The immune phenotypes and cytokine production were identified by FC500 flow cytometer (Beckman Coulter, USA) and Guava easyCyte™ flow cytometers (Merck Millipore, Germany). The monoclone antibodies were CD3+ to evaluate total T lymphocytes, CD3+/CD4+ to evaluate Helper T cells, CD3+/CD8+ to evaluate cytotoxic T cells (CTLs), CD3+/CD56+ to evaluate cytokine-induced killer (CIKs), CD4+/CD25+/CD127low/− to evaluate regulatory T cells (Tregs), CD80/CD86/CD83/CD11c to evaluate Dendritic cells (DCs), IFN-r to evaluate production of Interferon gamma and CD11b+/CD33+/HLA−DR− to evaluate Myeloid-derived suppressor cells (MDSCs) [17]. The proportion of Treg cells was calculated by the ratio of CD25hiCD127lo/− cells to CD4+ cells. The proportion of MDSC cells was calculated by the ratio of CD11b+ CD33+ cells to HLADR− cells. The flow cytometry data were analyzed by CXP software and guavaSoft™ 3.1.1.

### Detection of serological tumor markers by immunoradiometric methods

Serum AFP (Autobio, China), CEA (Roche, Switzerland), CA19-9 (Bayer, America), CA125 (Roche, Switzerland), CA242 (DPC, America) and CA724 (DPC, America) were detected by immunoradiometric methods with commercially available diagnostic kits, respectively. The recommended cutoff-values for diagnostic purpose were 20 μg/L for AFP, 5 μg/L for CEA, 37 U/ml for CA19-9, 35 U/ml for CA125, 12 U/ml for CA242 and 6 U/ml for CA742. Values above the cutoff concentrations were considered positive in this study.

### Evaluation and statistical analysis

Statistical analyses were completed using statistical SPSS 22 software (SPSS, Chicago, IL, USA). Analysis of biomarkers was measured using paired *t* test with P < 0.05 considered significant. The EORTC QLQ-C30 questionnaire [18, 19], developed by European Organization for Research and Treatment of Cancer (EORTC), is used to assess the quality of life of cancer patients. The hazard ratio was calculated by Cox’s proportional hazards regression method.

### Side effects

The criteria used to assess side effects included temperature, blood pressure, allergic reaction, appetite, fatigue and skin eruption.

## Results

### Characteristics of patients

We included patients with malignant tumors are not cured by traditional forms of therapeutic regimens such as surgery, radiation, or chemotherapy. The demographic characteristics of patients enrolled were shown in Table 1. Our analysis mainly used the data of cohort 1, in which 60 patients with high preoperative levels of the serum tumor markers have received at least two cycles of DC-CTL/CIK therapy. Fifty-nine patients with enough peripheral blood were available for analysis by flow cytometry, while one case was excluded for missing data. Median age of the patient was 54 years with a range of 27–81 years, 18 were female and 42 were male. The pathologic stage was Stage I in 3 patients (5 %), Stage II in 4 patients (6.7 %), Stage IIIA in 12 patients (20 %), Stage IIIB in 2 patient (3.3 %), Stage IIIC in 9 patient (15 %) and Stage IV in 27 patients (45 %). Among the 60 patients, 48 % (29/60) of the patients have received surgery before, and 40 % (24/60) of the patients received other adjuvant therapeutic strategies during the immunotherapy in which 18 (30 %) cases received chemotherapy, 8 (13.3 %) cases received radiation therapy, 2 (3.3 %) cases received physiotherapy and molecular targeted therapy, respectively, and other 9 (15 %) cases received interventional treatment. The detail information of cohort 2 and cohort 3 was also listed in Table 1. Furthermore, we subsequently performed univariate analyses by using the Cox proportional hazards regression model. Table 2 showed that there were no statistically significant differences between the two groups in terms of variables such as sex, age, TNM stage and receipt of adjuvant therapeutic strategies or not (P > 0.05), which ensured that our statistical analysis of immunotherapy efficacy was comparable.

**Table 1:** Characteristics of patients

Characteristic	Cohort 1 N (%)	Cohort 2 N (%)	Cohort 3 N (%)
No. of patients, N	60	14	9
Sex			
Male	42 (70 %)	8 (57.1 %)	6 (66.7 %)
Female	18 (30 %)	6 (42.9 %)	3 (33.3 %)
Age, median years (range)	54 (27–81)	59.5(40–76)	65 (42–82)
Tumor classification			
Cholangiocarcinoma	10 (16.7 %)	0	2 (22.2 %)
Colon cancer	7 (11.7 %)	1 (7.1 %)	0
Gastric cancer	7 (11.7 %)	0	0
Lung cancer	11 (18.3 %)	4 (28.6 %)	2 (22.2 %)
Primary liver cancer	25 (41.7 %)	1 (7.1 %)	2 (22.2 %)
Other	0	8 (57.1 %)	3 (33.3 %)
TNM staging			
Stage I	3 (5 %)	1 (7.1 %)	2 (22.2 %)
Stage II	4 (6.7 %)	0	1 (11.1 %)
Stage IIIA	12 (20 %)	2 (14.3 %)	1 (11.1 %)
Stage IIIB	2 (3.3 %)	0	0
Stage IIIC	9 (15 %)	4 (28.6 %)	1 (11.1 %)
Stage IV	27 (45 %)	6 (42.9 %)	3 (33.3 %)
Missing data	3 (5 %)	1 (7.1 %)	1 (11.1 %)
Adjuvant therapeutic strategies			
Without	24 (40 %)	5 (35.7 %)	7 (77.8 %)
With	36 (60 %)	9 (64.3 %)	2 (22.2 %)
Chemotherapy	18 (30 %)	6 (42.9 %)	1 (11.1 %)
Radiation therapy	8 (13.3 %)	1 (7.1 %)	1 (11.1 %)
Physiotherapy	2 (3.3 %)	0	1 (11.1 %)
Interventional treatment	9 (15 %)	2 (14.3 %)	0
Molecular targeted therapy	2 (3.3 %)	1 (7.1 %)	0

**Table 2:** Univariate analysis in patients

Variables	OR (95 % Cl)	P
Sex (male vs. female)	0.880 (0.360–2.153)	0.779
Age (≤54 vs. >54)	1.003 (0.411–2.447)	0.995
TNM stage (I, II vs. III, IV)	1.310 (0.297–5.777)	0.722
Chemotherapy (no vs. yes)	0.419 (0.160–1.100)	0.077
Radiation therapy (no vs. yes)	0.643 (0.177–2.337)	0.502
Surgery (no vs. yes)	0.983 (0.398–2.430)	0.971
Molecular targeted therapy (no vs. yes)	0.671 (0.123–3.659)	0.645
Physiotherapy (no vs. yes)	0.594 (0.044–7.968)	0.694
Interventional treatment (no vs. yes)	0.615 (0.174–2.171)	0.450

*CI* confidence interval

### Quality control and phenotypes of infused cells

**Fig. 1:**
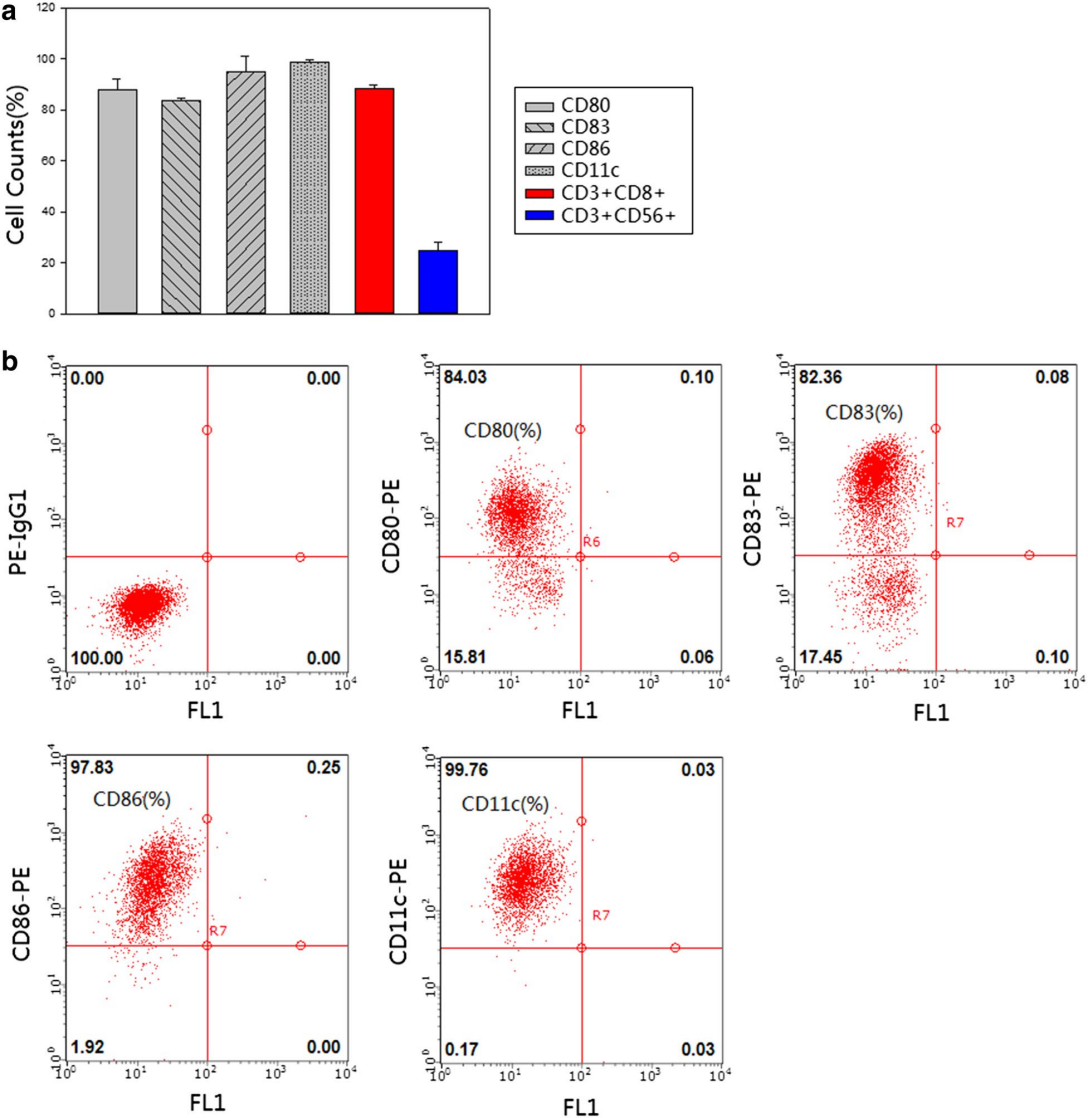
Analysis of the percentage of cell markers. **a** The histogram showed the percentage of each marker. The *X axis* represented cell markers of CD80, CD83 CD86 CD11c, CD3+ CD8+ and CD3+ CD56+ respectively, and the proportion of positive cells was calculated that was represented on *Y axis* [cell counts (%)]. The standard deviation was also labeled on the histogram. **b** On Day 8, DCs were harvested and processed for staining with CD80+, CD83+, CD86+ and CD11c antibody respectively. The surface marker was identified by flow cytometry and the percentage of positive cells was stated. The first plot represented Isotype group

**Fig. 2:**
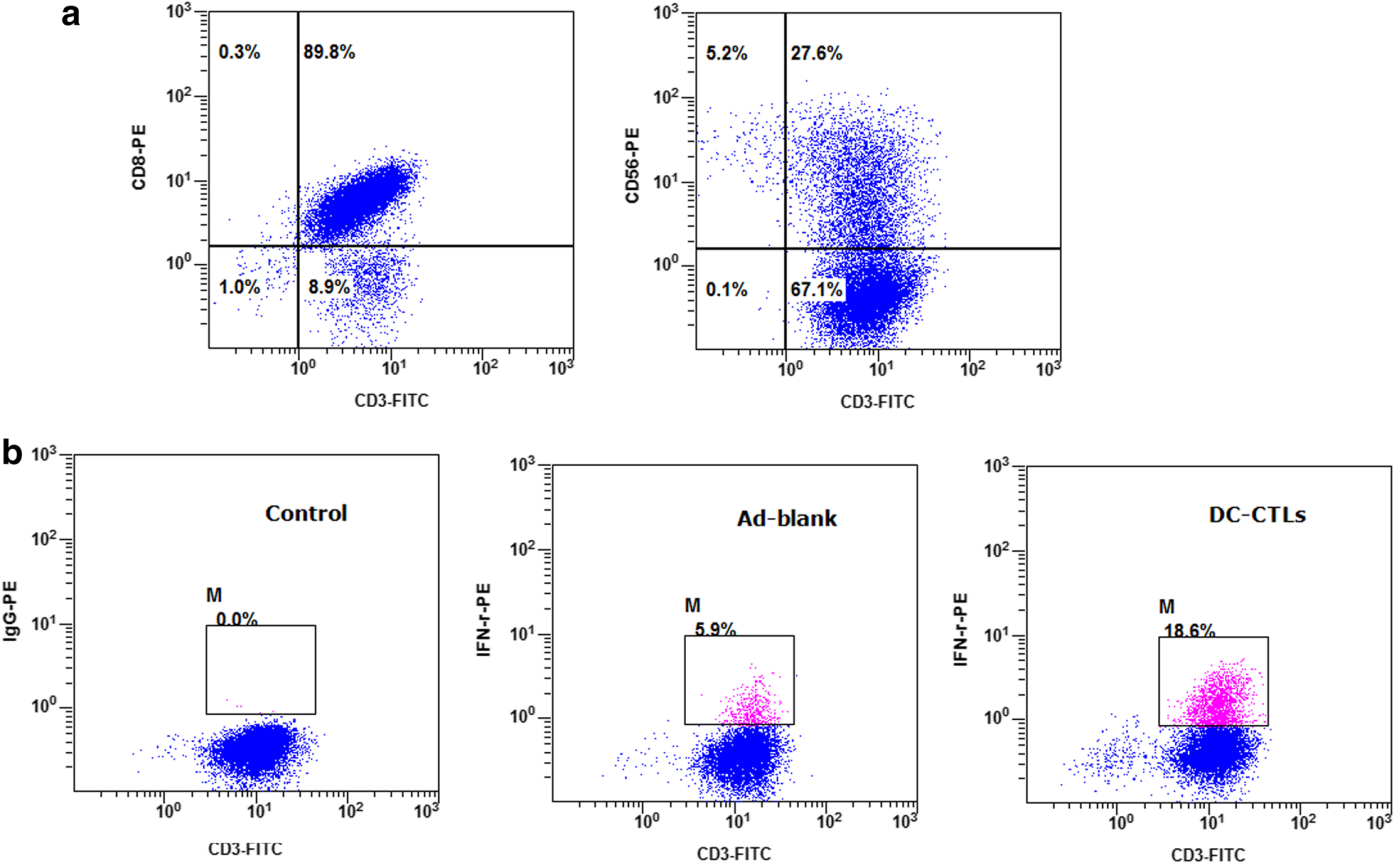
Detection of CIKs, CTLs and intracellular IFN-γ in T cells by flow cytometry. **a** On Day 10, the proportions of CD3+ CD56+ T cells were used to detect cytokine-induced killer cells (CIKs). And on Day 12, the proportion of CD3+ CD8+ T cells was used to identify cytotoxic T-lymphocyte cells (CTLs). The position of the gate was set by Isotype control. **b** The *dot* *plots* showed the proportion of T cells expressing IFN-γ. The *left plot* represented isotype control group, the *middle plot* represented Ad-Blank group, and the *right plot* represented the production of cytokines by DC induced antigen-specific CTL cells, and DC cells were infected by the recombinant non-proliferative adenovirus carrying the expression cassettes of TAAs

DCs immunophenotype changes were detected by flow cytometry at Day 8. As show in Fig. 1a, it exhibited high-level expression of CD11c (98.71 ± 0.93 %), costimulatory molecules CD80 (86.55 ± 4.36 %), CD86 (95.11 ± 6.13 %) as well as the maturation marker CD83 (83.65 ± 1.12 %), which confirmed the maturation of DC cells after cultivation. Remarkably, the proportion of CD3+ CD56+ cells in vitro incubation reached 24.97 ± 3.13 % at Day 10. The percentage of cytotoxic T-lymphocytes (CTL, CD3+ CD8+) at day 12 reached 88.19 ± 1.43 %. The plot of flow cytometry was also shown in Figs. 1b and 2a. Notably, IFN-γ exhibited high production during in vitro cultivation, and the proportion of T cells expressing IFN-γ was determined to be 18.6 % on Day 12, compared to Ad-blank group (Fig. 2b). Additionally, the infused cells were cultured for the detection of bacteria, fungus and endotoxin levels, all of which met the release criteria.

### Individual analysis of the changes of immune indicators and serological tumor markers pre- and post-treatment

**Fig. 3:**
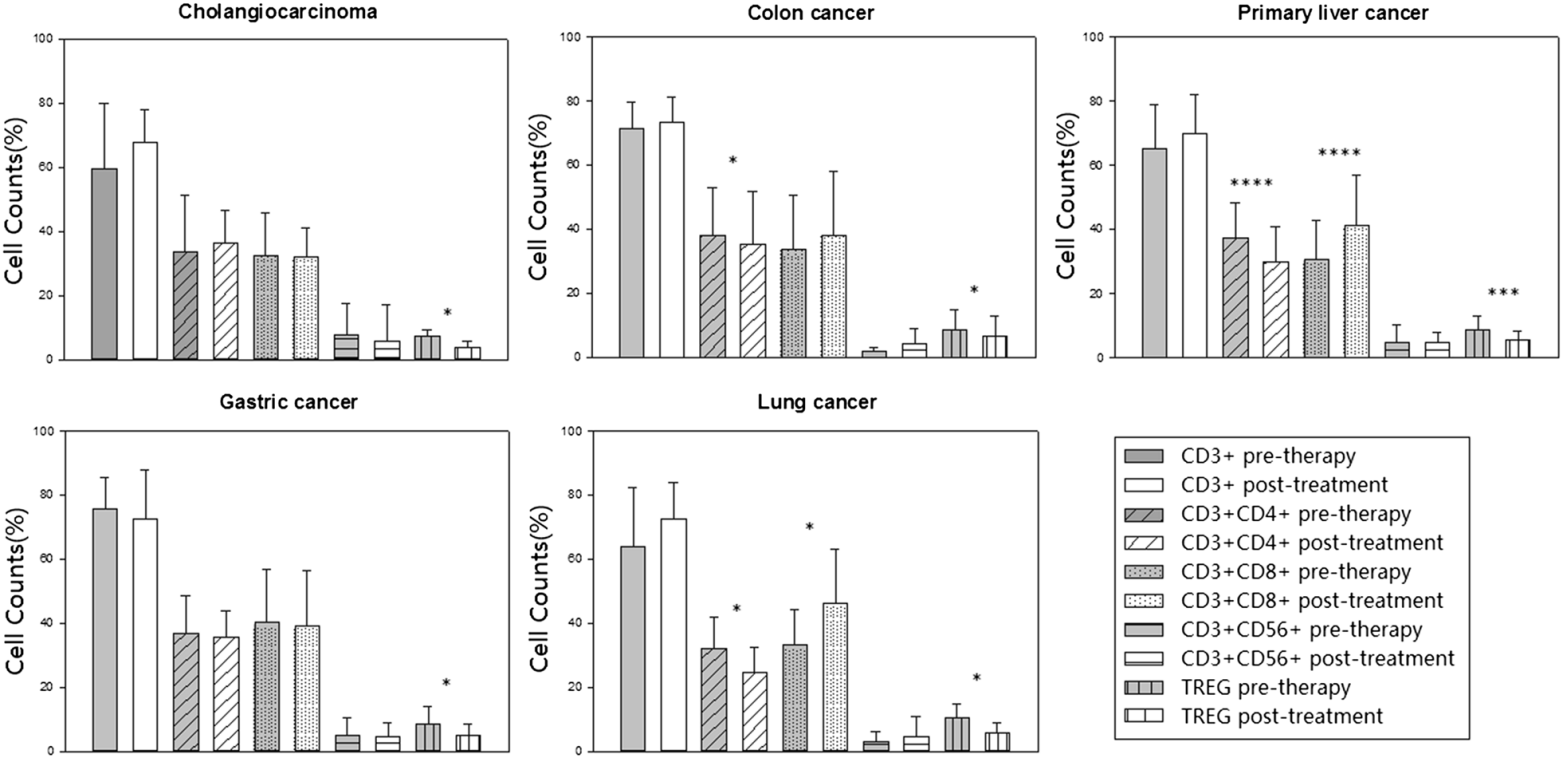
Changes of immune indicators pre- and post-treatment in five types of tumors. The changes of CD3+, CD3+ CD4+, CD3+ CD8+, CD3+ CD56+ and Treg in patients with cholangiocarcinoma, colon cancer, primary liver cancer, gastric cancer and lung cancer pre-therapy and post-treatment, respectively. *Statistically significant differences (*p < 0.05, **p < 0.01, ***p < 0.005, ****p < 0.001)

We firstly observed the changes of serological tumor markers and immune indicators pre-therapy and post-treatment (defined as their most recent course of DC-CTL/CIK treatment). Notably, the percentage of Treg cells in peripheral blood significantly decreased post-treatment (p < 0.05) in all tumor types (Fig. 3). This was a very encouraging observation, considering Treg as an immunosuppressive cell could induced immune escape and suppressed anti-tumor immune response. We also found that the proportion of CD3+ CD4+ T cells decreased significantly in colon cancer, primary liver cancer and lung cancer (p < 0.05), while the percentage of CD3+ CD8+ T cells increased significantly in primary liver cancer and lung cancer (p < 0.05). Among the 6 common tumor markers, the serum levels of AFP, CA19-9 and CA242 post-treatment were significantly lower than that of pre-therapy in primary liver cancer (p < 0.05). The serum levels of CA724 also decreased significantly, which is often used in the diagnosis of gastric cancer (p < 0.05). Additionally, there were 8 and 13 patients showed declined levels of CEA and CA125 in serum, respectively, in which 4 and 8 patients returned to normal levels post-treatment, but the difference was not statistically significant (p > 0.05) (Additional files 1, 2: Figures S1, S2).

### Systematic analysis of correlation between proportions of Tregs and prognosis

**Fig. 4:**
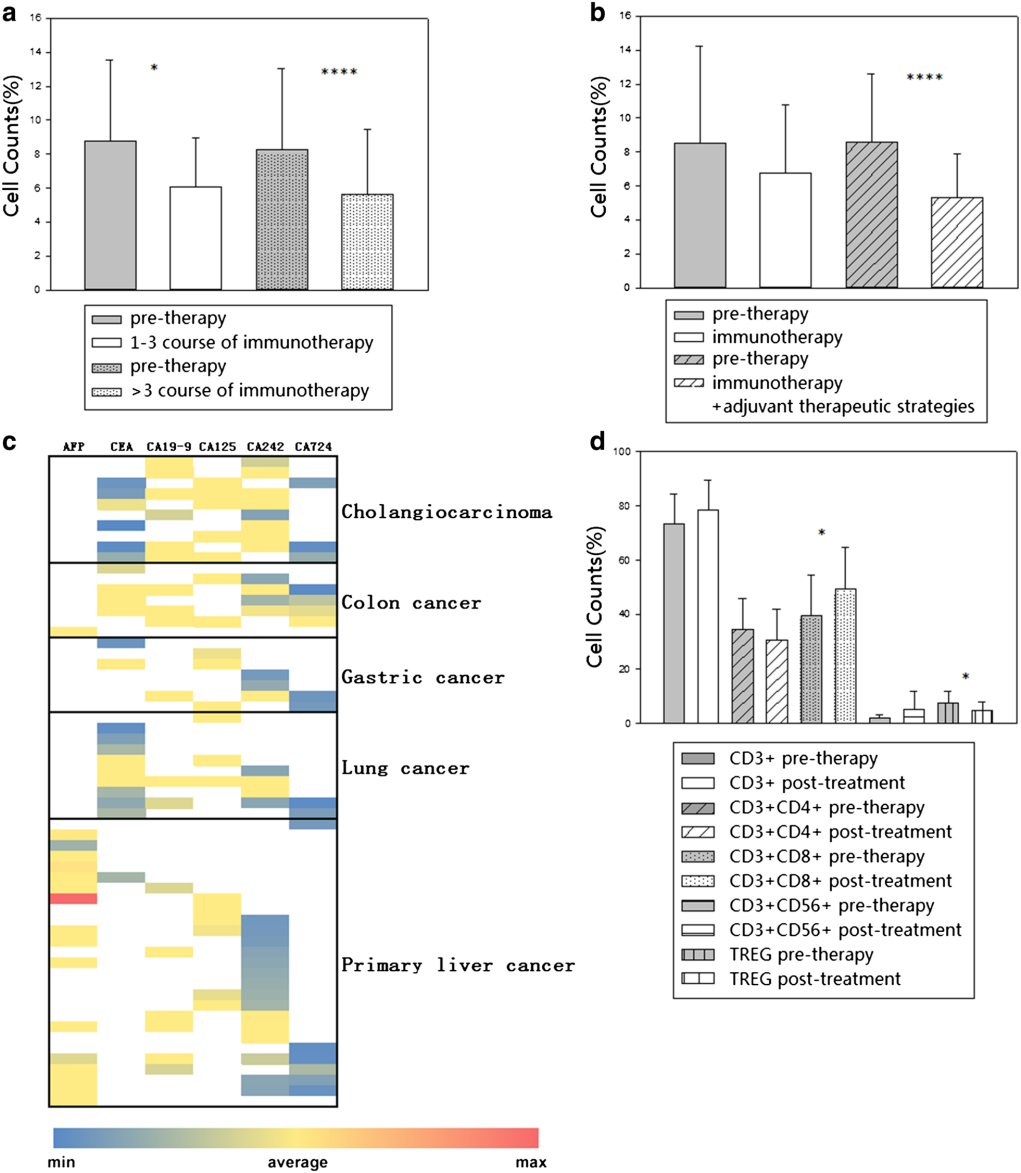
Analysis of correlation between proportions of Tregs and prognosis. **a** The changes of percentages of Treg cells in patients with short or long-term courses of immunotherapy, compared to pre-therapy group. The *dot-bar* represented patients enrolled in immunotherapy more than three cycles. **b** The changes of percentages of Treg cells in patients with immunotherapy alone or in combination with other adjuvant therapeutic strategies, compared to pre-therapy group. The *diagonal-bar* represented patients recruited during our immunotherapy combined with other adjuvant therapies. *Statistically significant differences (*p < 0.05, **p < 0.01, ***p < 0.005, ****p < 0.001). **c** The heatmap showed the clinical values of six serum tumor markers for each type of tumor. *Each* *color* represented the level of tumor markers in serum, and the *color* legend was shown at the *bottom* of the heatmap. **d** The changes of immune indicators including CD3+, CD3+ CD4+, CD3+ CD8+, CD3+ CD56+ and Treg in 14 patients who were with negative for all serological indexes. *Statistically significant differences (*p < 0.05)

Subsequently, a systematic analysis was performed to explore the changes of proportions of Tregs on the basis of treatment courses and therapeutic strategies. Notably, it demonstrated that long-lasting course treated patients (>3 times) had lower percentages of Tregs (5.65 ± 3.80 %) than patients who only received short-term therapy (6.07 ± 2.91 %), and proportion of Tregs reduced more significantly in patients with long-lasting course treatment (p = 0.0009) than those with short-term therapy (p = 0.01) (Fig. 4a). Moreover, Treg indicator was also observed in patients with immunotherapy alone or in combination with adjuvant therapies, indicating that the percentage of Treg decreased for these patients with immunotherapy alone (6.77 ± 4.04 %) or combined with other adjuvant therapeutic strategies (5.3 ± 2.58 %), and it significantly decreased after combination of DC-CTL/CIK therapy and adjuvant therapeutic strategies (p = 0.00005) (Fig. 4b). Then, the Cox’s proportional hazards regression was further performed between patients with low (response) and high (non-response) proportions of Tregs. The hazard ratio was 2.021, indicating higher hazard of death in non-response group.

Additionally, a heat map was performed to describe by each six indicators of cancer to compare the difference between different types of cancer pre-therapy. As shown in Fig. 4c, in fact, it is difficult to find a single marker that has high sensitivity and specificity to achieve clinical utility. An elevated level of a tumor marker is different in many kinds of tumors. In our study, cases in Cohort 1 were positive for one of six tumor markers, the percentage of Treg cells significantly decreased post-treatment in patients with all types of tumors. Thus, we further observed other 14 cases in Cohort 2 that were negative for all six tumor markers pre-therapy. Interestingly, the changes of immune indicators (Fig. 4d) were basically consistent with those of tumor marker positive cases, showing that the percentage of CD3+ CD8+ T cells increased and Tregs reduced significantly post-treatment. Thus, we demonstrated that the percentage of Tregs was significantly correlated with the survival time of tumor patients and a combination of serological tumor markers and Treg to investigate the therapeutic roles of DC-CTL/CIK therapy could be more efficiently in the patients with malignant tumors.

### Assessment of quality of life (QoL)

We measured quality of life of patients undergoing DC-CIK/CTL treatment. The QLQ-C30 has been suggested to have multi-item subscales measuring function and symptom. A quality-of-life feature, QoL score, was designed to evaluate the balance between side effects and quality of life. It is inspiring that the QoL score of 25 (41.7 %) patients improved obviously post treatment, and 12 (20 %) patients did not take a turn for the worse. Thus, it indicated that DC-CIK/CTL therapy could at least partially block the deterioration of cancers in the patients with malignant tumors with elevated quality of life.

### Comparison of MDSC levels pre- and post-treatment of DC-CTL/CIK therapy

**Fig. 5:**
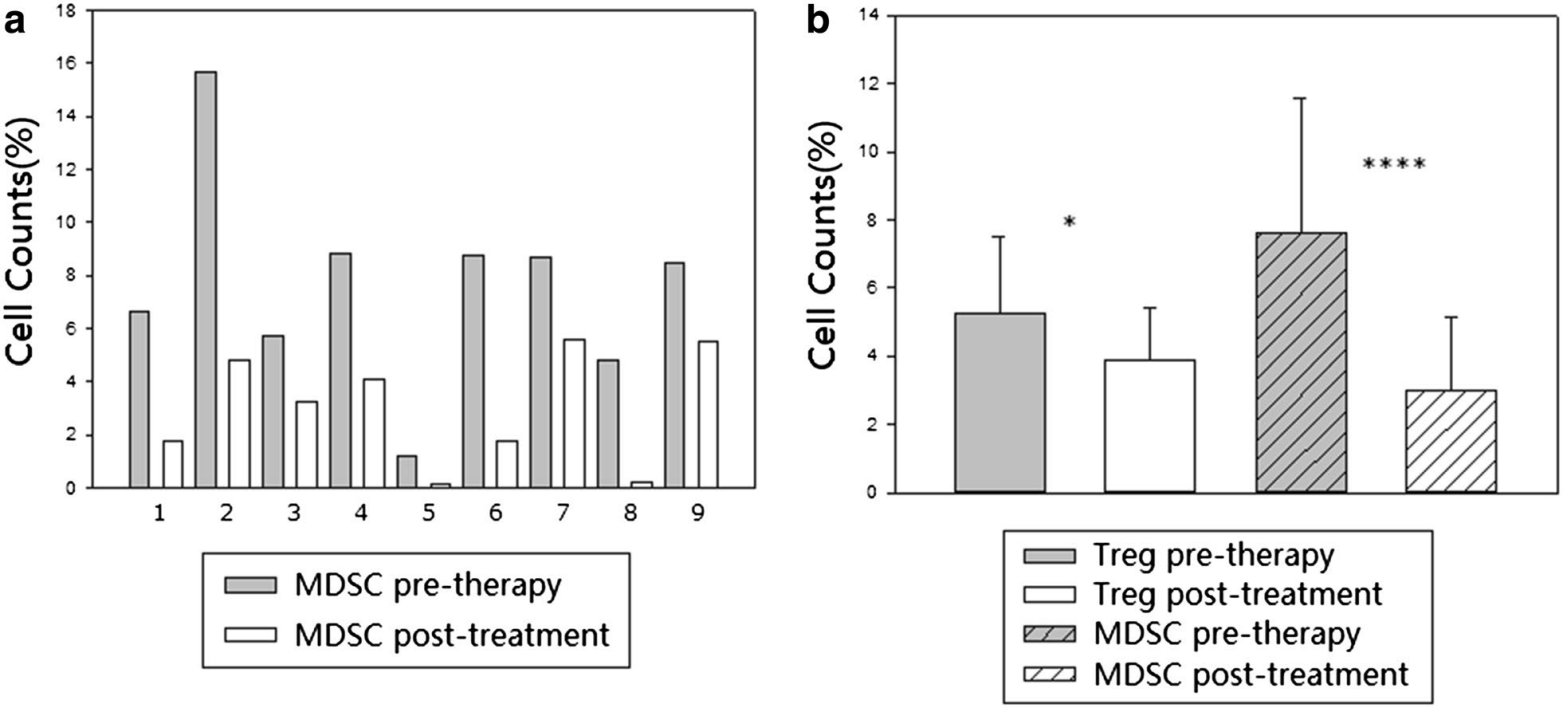
Significance of MDSC populations in peripheral blood. **a** The changes of MDSC levels pre- and post-treatment of DC-CTL/CIK therapy in each nine patient (cohort 3). **b** The histogram showed the percentage of Treg cells and MDSC cells in cohort three patients pre-therapy and post-treatment. *Statistically significant differences (*p < 0.05, **p < 0.01, ***p < 0.005, ****p < 0.001)

To analyze the changes of MDSCs in DC-CTL/CIK therapy, other nine patients were included for the detection of MDSCs and Tregs. The results showed a significant decrease in the proportion of MDSC and Treg after treatment (p = 0.000681, p = 0.035, Fig. 5), which provided an additional proof of therapeutic effect of DC-CTL/CIK therapy. Further studies will be needed to confirm this preliminary result in our future work.

### Side effects

The side-effects were also recorded in all cases. There were no severe or unusual side-effects recorded that are common in chemotherapy and radiotherapy during or after the transfusion, except for temporary fever. In total, 40 % patients developed a fever during the immunotherapy, and the body temperature was 37.5–38. These patients recovered without any treatment. Six out of 60 (10 %) cases had temperatures >38 °C treated with drugs to lower body temperature.

## Discussion

In this study, we collected three cohorts of patients to investigate therapeutic effect and immune modulation of DC-CTL/CIK cells in malignant tumors both at an individual level (changes of immune indicators and serological tumor markers pre- and post-treatment) and at a system level (correlation between proportions of Tregs and prognosis). We detected several serological tumor markers and immune indicators pre-therapy. Levels of 6 common tumor markers in 60 cases were highly expressed in serum. The abnormal level of T lymphocyte subpopulations indicated severe damage of immune systems in these patients, and the accumulation of Tregs within the tumor microenvironment represents a major obstacle for the development of effective antitumor immune-therapies [16, 20]. Excitingly, we found out that our DC-CTL/CIK therapy significantly reduced several serological tumor markers such as AFP, CA199 and CA242 in primary liver cancer and CA724 in gastric cancer (p < 0.05), elevated the level of CD3+ CD8+ T cells in primary liver cancer and lung cancer (p < 0.05), and decreased the level of CD3+ CD4+ T cells in colon cancer, primary liver cancer and lung cancer (p < 0.05) and Treg cells in all types of tumors (p < 0.05), which indicated the promotion of immune functions in these patients. The quality of life (QoL score) of patients was improved after treatment which could be indicate that it exerts immune and clinical responses in patients with malignant tumors DC/CIK treatment.

A systematic analysis was performed to discuss the correlation between proportions of Tregs and prognosis, and whether a combination of immune indicators especially Treg indicator with serum tumor markers could be a better way to evaluate therapy responses. Tregs within tumors represented a major obstacle for cancer immunotherapy [21, 22]. In previous studies, it has been reported that Tregs decreased the cytotoxicity of CIK cells, and the down-regulation of Tregs could strengthen the killing activity of CIK cells to tumor cells [14, 16, 23]. Tao [16] and Lin [23] indicated that the addition of IL-6, IL-7, IL-15 during CIK cell culture in vitro inhibited the production of Tregs. Pan [14] suggested that DC cells decreased concomitant expanded Tregs and Tregs related IL-35 in CIK cells, to against leukemia cell lines K562 and NB4.

Here, compared with the prior methods, we also provided a novel cell culture program in the yield of DC cells, a cocktail of antigens and incubation time, to significantly reduce the proportion of Tregs. As in our cell culture protocol, it has greatly reduced the percentage of Tregs (less than 7 %). Analysis at a system level showed that long-lasting course of DC/CIK treated patients (>3 cycles) had lower percentages of Tregs than patients who only received short-term therapy, and the percentage of Treg was lower for these patients with immunotherapy alone or combined with other adjuvant therapeutic strategies.

Moreover, to date, the detection of tumor markers has been widely used in cancer screening, early diagnosis, monitoring efficacy of treatment, and prognostic evaluation [24–27]. In fact, it is difficult to find a single marker that has high sensitivity and specificity to indicate a cancer. For instance, alpha-fetoprotein (AFP), a widely accepted biomarker of hepatocellular carcinomas (HCC), in some patients with advanced HCC may still be negative. AFP in our dataset was elevated in 94 % of liver cancer patients, but 10 cases were AFP-negative. As such, it is likely that the effect of DC-CTL/CIK immunotherapy would not be suitably assessed by methods based on response evaluation criteria in solid tumors (RECST). Therefore, it is urgently necessary to identify and validate reliable biomarkers to evaluate clinical responses and outcomes of immunotherapy including DC-CTL/CIK therapy. A methodology named “Immunoscore”, which numerates lymphocytes both in tumor center (CT) and invasive margin (IM), has been introduced to add to the significance of the American Joint Committee on Cancer (AJCC)-Union for International Cancer Control (UICC)-Tumor Node Metastasis (TNM)-classification, and to establish prognosis of clinical outcome in patients [28]. In our study, we proposed that altered immune phenotype in peripheral blood cells of patients may have clinical value in the evaluation of therapeutic effects and clinical outcome, especially Treg cell as a potent immunosuppressive cell which promote tumor growth and invasion. MDSC also has been proposed to have contribution to immune-suppression, and have association with grades, stages, tumor burden and clinical outcomes in patients with different types of cancer [29]. MDSCs can influence the proliferation and activation of T cells, NK cells, dendritic cells and macrophages, especially in inhibiting CD8+ T cell responses [30–32]. Diaz-Montero [33] demonstrated that MDSC can be referred to as an independent prognostic indicator by multivariate Cox proportional hazards analysis. Here, we also perform a preliminary investigation of MDSCs in peripheral blood pre and post-therapy, and more verification experiments are needed to undertake this improvement in the future.

In addition, as many papers have published that neo-antigens in cancer immunotherapy and personalized antigen for the treatment [34], optimization of this program will be further performed to standardize and facilitate the cell culture process. Besides DC-CTL/CIK immunotherapy in this study, other immunotherapeutic agents also have made great progress in clinic, such as immune checkpoint inhibitor antibodies of PD-1/PD-L1 or CTLA-4, and chimeric antigen receptor-T cell (CAR-T) targeting specific tumor antigen. CTLA-4 antibody ipilimumab, PD-1 antibody pembrolizumab and nivolumab have been approved by FDA [35]. The most successfully application of CAR-T is on chronic lymphocytic leukemia patients, which specifically binds to CD19 marker. However, immunosuppressive microenvironment in tumor (surrounding with Treg, MDSC, etc.) may greatly impaired the therapeutic effects of immune checkpoint inhibitors and CAR-T. Moreover, there are rare target antigens for T cell engineering [10, 36]. Our DC-CTL/CIK immunotherapeutic agents can greatly improve the system of immune function and reduce Tregs and MDSCs in peripheral blood, and also with widely antitumor spectrum of solid tumors. Furthermore, combination of DC-CTL/CIK with immune checkpoint inhibitors or CAR-T may bring out better clinical result, and the clinical trials are being undertaken in our hospital which is believed to greatly improve the therapeutic effect of immunotherapy in the future.

In summary, our results demonstrated that DC-CTL/CIK therapy as an immunotherapeutic regimen could influence the immune status, and patients with late-stage malignant tumor may benefit from this therapeutics. Our data conclusively exhibited immune improvement by impairing the immune-suppressive Tregs after DC-CTL/CIK therapy. This immune response is continuously modulated with the increasing of treatment courses. We have also showed a combination of DC-CTL/CIK therapy with other adjuvant therapeutic strategies could reduce the percentages of Treg significantly, and a decrease frequency and depleted suppressive activity of Tregs in peripheral blood in patients which have a strong correlation with prognosis. Thus, this work may provide valuable insights into the clinical curative effect evaluation of DC-CTL/CIK therapy and the design of immunotherapeutic strategies for malignant tumors.

## Additional files

10.1186/s40164-015-0027-9 Changes of serum level of AFP and CEA pre- and post-treatment in five types of tumors. Analysis of the changes of AFP and CEA in patients with cholangiocarcinoma, colon cancer, primary liver cancer, gastric cancer and lung cancer pre-therapy and post-treatment, respectively. *Statistically significant differences (*p < 0.05).
10.1186/s40164-015-0027-9 Changes of serum level of tumor marker carbohydrate antigen pre- and post-treatment in five types of tumors. Analysis of the changes of CA199, CA724, CA125 and CA242 in patients with cholangiocarcinoma, colon cancer, primary liver cancer, gastric cancer and lung cancer pre-therapy and post-treatment, respectively. *Statistically significant differences (*p < 0.05).

## Abbreviations

DC: dendritic cells
CIK: cytokine-induced killer
CTL: cytotoxic T lymphocytes
MDSC: myeloid-derived suppressor cells
PBMCs: peripheral blood mononuclear cells
EORTC: European Organization for Research and Treatment of Cancer
QoL: quality of life
AFP: Alpha-fetoprotein
HCC: hepatocellular carcinomas
CT: tumor center
IM: invasive margin
AJCC: American Joint Committee on Cancer
UICC: Union for International Cancer Control
TNM: tumor node metastasis
