# Supplementary figures and images for: The combination of dendritic cells-cytotoxic T lymphocytes/cytokine-induced killer (DC-CTL/CIK) therapy exerts immune and clinical responses in patients with malignant tumors
Source: Exp Hematol Oncol. 2015 Nov 10;4:32. doi: 10.1186/s40164-015-0027-9 (PMC4641330; doi:10.1186/s40164-015-0027-9)

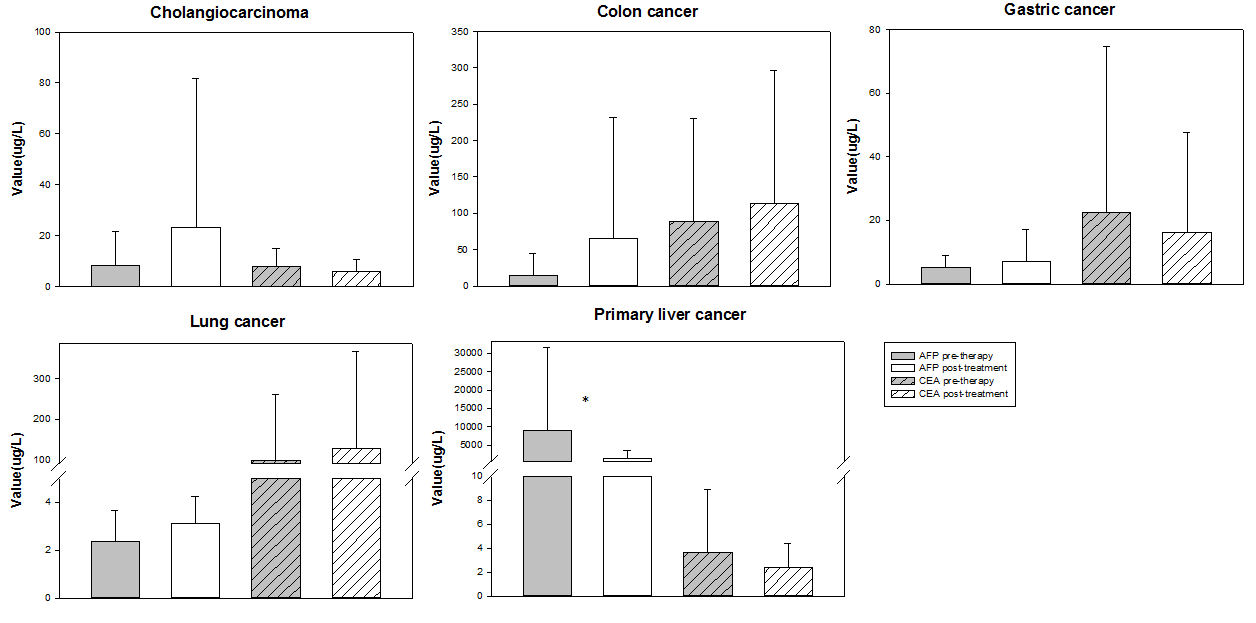

Supplement: Supplementary file 1 — 10.1186/s40164-015-0027-9 Changes of serum level of AFP and CEA pre- and post-treatment in five types of tumors. Analysis of the changes of AFP and CEA in patients with cholangiocarcinoma, colon cancer, primary liver cancer, gastric cancer and lung cancer pre-therapy and post-treatment, respectively. *Statistically significant differences (*p < 0.05). [file 40164_2015_27_MOESM1_ESM.png]

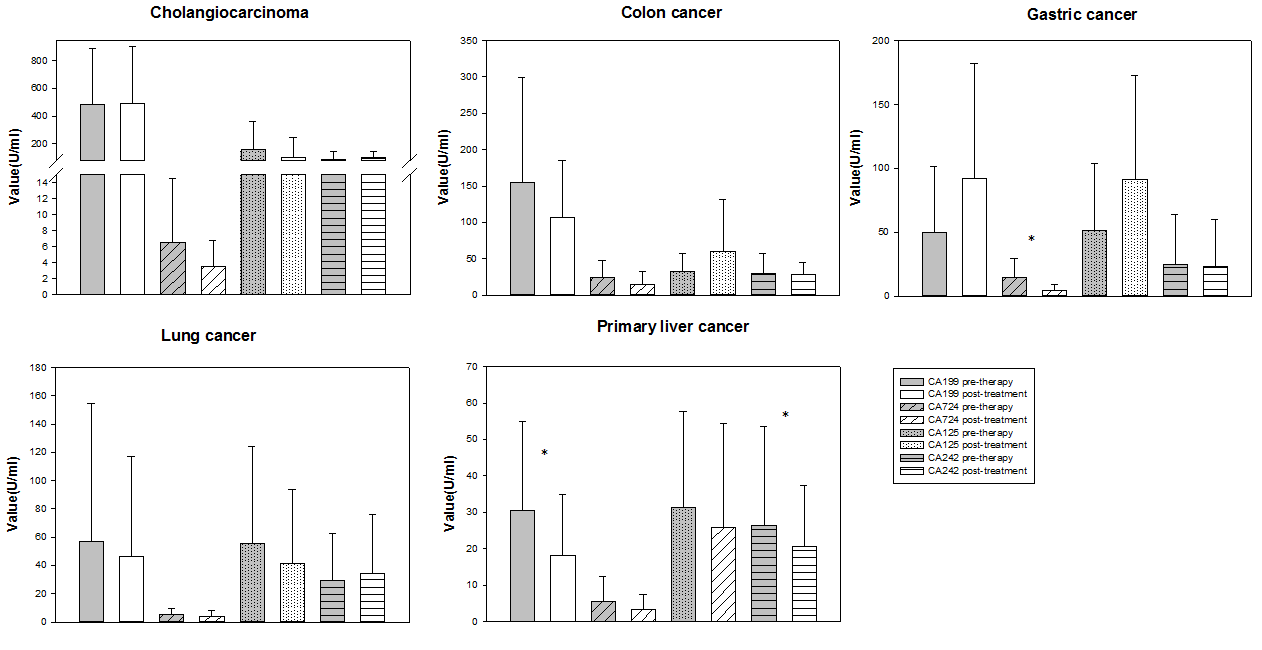

Supplement: Supplementary file 2 — 10.1186/s40164-015-0027-9 Changes of serum level of tumor marker carbohydrate antigen pre- and post-treatment in five types of tumors. Analysis of the changes of CA199, CA724, CA125 and CA242 in patients with cholangiocarcinoma, colon cancer, primary liver cancer, gastric cancer and lung cancer pre-therapy and post-treatment, respectively. *Statistically significant differences (*p < 0.05). [file 40164_2015_27_MOESM2_ESM.png]
